# Respiratory disease and sero‐epidemiology of respiratory pathogens in the working horses of Ethiopia

**DOI:** 10.1111/evj.12834

**Published:** 2018-05-17

**Authors:** G. Laing, R. Christley, A. Stringer, N. Aklilu, T. Ashine, R. Newton, A. Radford, G. Pinchbeck

**Affiliations:** ^1^ Institute of Infection and Global Health University of Liverpool Liverpool UK; ^2^ SPANA (Society for the Protection of Animals Abroad) Debre Zeit Ethiopia; ^3^ Animal Health Trust Newmarket Suffolk UK

**Keywords:** horse, strangles, *Streptococcus equi*, prevalence, serology, Africa, ELISA

## Abstract

**Background:**

Pathogens are frequently implicated in equine respiratory disease. In Ethiopia, respiratory disease is a frequent cause for presentation at veterinary clinics and a priority concern for users of working horses. However, there is little existing literature on possible aetiologies.

**Objectives:**

Determine prevalence of respiratory signs and exposure to major respiratory pathogens through a serological survey.

**Study design:**

Cross‐sectional.

**Methods:**

Systematically selected horses from 19 sites in central Ethiopia were examined clinically and sampled once (August‐December 2013). A face‐to‐face interview collected data on horses’ management and history. Serological testing targeted equine influenza virus (EIV), equine herpesviruses‐1 (EHV‐1) and ‐4 (EHV‐4), equine rhinitis viruses A (ERAV) and B (ERBV), equine arteritis virus (EAV) and *Streptococcus equi* subspecies *equi* (*S. equi*).

**Results:**

Owners reported a recent history of coughing in 38% of horses and nasal discharge in 7%. No animals were observed coughing during examination but 6% had a nasal discharge. Antibodies towards *S. equi*, were most prevalent (8%, 33/350). Antibodies to EAV were confirmed in one animal (0.3%). Low antibody titres to EHV‐1/4 and ERA/BV suggested prior exposure but antibodies to EIV were not detected. Multivariable, multilevel logistic regression analysis for risk factors associated with *S. equi* serostatus showed higher odds of seropositivity in younger animals and those working less frequently.

**Main limitations:**

A single serological sample cannot describe dynamic changes in antibodies. Sampling horses at the place of work may result in healthy‐worker bias.

**Conclusions:**

*S. equi* may be endemic in this population and contributing, in part, to the occurrence of respiratory disease. Low prevalence of antibodies to viruses, with the exception of EIV, indicates these pathogens are present, but unlikely a predominant cause of respiratory signs and noninfectious causes of disease should also be investigated. Working horses in this region would be vulnerable to incursion of equine influenza.

## Introduction

Ethiopia has Africa's largest equid population which is continuing to grow 1. The 2 million working horses are used in urban and rural environments to pull cart‐taxis as ‘gharry’ horses or ridden as saddle horses. They transport people, produce, water and building materials and are an important source of income for whole families 2.

Respiratory disease is recognised as impacting work ability in equines worldwide 3, 4, 5, with coughing and nasal discharge, being a priority concern for users of working horses in Ethiopia 2, 5, 6. A recent assessment of working equid health 7 identified a poorly defined respiratory syndrome as one of three syndromes with high prevalence and morbidity and that investigation to determine the role of infectious agents is critical. In addition, respiratory conditions identified at SPANA (Society for the Protection of Animals Abroad) veterinary clinics over a 5‐year period accounted for up to 15% of all horses seen, including those presented for routine veterinary interventions such as vaccination or deworming (2007–2012; unpublished data). Despite this, there is little literature on the possible aetiology of respiratory disease in Ethiopia or other countries with a large working equid population. Elsewhere infectious causes of respiratory disease are common 8, 9 but diagnostic capabilities in Ethiopia mean prevalence data for major respiratory pathogens are lacking. As such, it is difficult to speculate as to the aetiology of disease or provide evidence‐based recommendations for treatments or preventive measures.

The objectives of this study were to determine the prevalence of respiratory signs in Ethiopian working horses and seroprevalence to major respiratory pathogens based on serological assays. Major respiratory pathogens considered were: equine influenza virus (EIV), equine herpesviruses‐1 (EHV‐1) and ‐4 (EHV‐4), equine rhinitis viruses A (ERAV) and B (ERBV), equine arteritis virus (EAV) and the bacterial pathogen *S. equi*.

## Materials and methods

A cross‐sectional study was conducted across 19 sites in central Ethiopia (August–December 2013). Sites included rural and urban locations with substantial horse populations across lowland (<1500 m) to highland (>2300 m) regions representative of the region. Informed consent was sought verbally from owners/drivers.

As literature for respiratory pathogen seroprevalence in other countries vary widely (1–95%) 8, 9, 10, 11, 12, a 50% prevalence was used to estimate sample size with an absolute precision of 10% and 95% confidence intervals 13. This was then adjusted for clustering of horses within towns according to Dohoo *et al*. 14. There was no existing literature on intra‐cluster correlation (ICC) for respiratory infections in Ethiopian horse populations so a best estimate of 0.1 was used based on ICC for infectious diseases in other species 15, 16. A cluster size of 18 provided a final sample size of 262 animals across 15 towns.

Horses, with their owner or driver, were selected from a focal point in the town such as a cart‐taxi waiting area or parking area at a market. A systematic sampling strategy was employed where every other horse was selected from a dynamic queue or parking place.

A brief questionnaire underwent reverse translation before being administered individually by an Ethiopian research assistant in either of two regional languages (Amharic or Afan Oromo). This included horse details, husbandry practices and history of respiratory signs in the preceding 30 days (Supplementary Item 1). Any history of respiratory disease was described by participants in their own words first, and if nasal discharge (ND) was volunteered, an illustrated chart developed during a previous participatory study 17 was used (Supplementary Item 2).

A brief but systematic clinical examination included jugular blood samples, an estimation of age based on dentition, body condition score (BCS, scale 0–5) 18, description of ND and breathing characteristics, thoracic auscultation, cranial lymph node assessment, and rectal temperature. Also recorded was information on harness fit where this was a possible cause for respiratory obstruction.

### Clinical samples

Serum was heat treated at 56°C for 30 min, then stored at 4°C until transported to the UK and stored at −20°C. Manual packed cell volume (PCV) and total plasma protein (TPP) were measured.

All serum samples were processed and analysed by the first author at the Animal Health Trust, Newmarket, UK (AHT). Serological tests were run in accordance with the laboratory's Standard Operating Procedures, with the exception of the commercially available influenza kit, which was conducted according to the manufacturer's guidelines^1^. All samples were run in duplicate with positive and negative controls on each plate. Table 1 summarises the serological methods used. ELISA optical density (OD) values were considered relative to both positive and negative controls. Samples achieving or exceeding the screening threshold OD in the EAV ELISA were subject to virus neutralisation test (VN) to identify true EAV seropositive results 19. Controls for cross‐reaction between test antigens and sample antibodies to all four viruses (EHV‐1, EHV‐4, ERAV and ERBV) on complement fixation tests were in place and any anticomplementary activity of serum samples considered when reporting final antibody titres.

**Table 1:** Serological tests used in this study and information on previously published methods, test sensitivity and specificity and criteria used for positive cutoff

Pathogen	Serological test	Published methods	Sensitivity/Specificity	Criteria for positive cutoff
*S. equi*	Dual antigen A & C ELISA	25	93.3%/99.3%	Positive: OD ≥0.5
Equine arteritis virus	ELISA	39	96.8%/95.6%	OD ≥ 0.5
	Virus neutralisation	40		Neutralisation ≥75%
Equine herpesvirus (1/4)	Complement fixation	35		Reciprocal dilution titre ≥1:80
Equine rhinitis virus (A/B)	Complement fixation	35		Reciprocal dilution titre ≥1:80
Equine influenza virus	ELISA	20	95.3%/79–99%	Percentage inhibition ≥55%
	Haemagglutination inhibition	41		Reciprocal dilution titre ≥1:32

OD, optical density; ELISA, enzyme‐linked immunosorbant assay; OIE, World Organisation for animal health, equine influenza virus strains for haemagglutination inhibition: H7N7 Prague/56, H3N8 Miami/63 and H3N8 Newmarket/2/93.

As no equine influenza strain information exists for Ethiopia, serology for EIV used a multispecies Influenza A competitive ELISA^1^ ^20^, ^21^. Manufacturer's guidelines were adapted with an initial sample/buffer mixing step and a strong and weak positive control from AHT library serum (H7N7 Prague/56, H3N8 Miami/63, H3N8 Newmarket/2/93). Any samples showing percentage inhibition <100% were subsequently tested using the OIE gold standard haemagglutination inhibition (HI) for known equine influenza strains.

### Data analysis

Due to the clustering of animals by sampling locations in a town, prevalence for serostatus to all pathogens was adjusted for clustering with estimates derived from intercept‐only models with sample location included as a random effect. Confidence intervals (95%) were calculated as a function of the standard error.

Horse‐ and owner‐level associations with serostatus were investigated. Associations with *S. equi* serostatus were examined using univariable and mulitvariable multilevel logistic regression models with a binomial distribution and logit link function in which sampling location (town) was included as a random effect using R. Serological test results were considered as a binary outcome (seropositive/seronegative) (Table 2). Continuous variables were assessed for linearity using generalised additive models (GAM) and then analysed using univariable logistic regression. Correlation between variables to be included in the multivariable logisitic regression model was assessed, with exclusion of correlated variables (correlation coefficient >0.7) with the greater P‐value. In building the final model all explanatory variables with a P‐value <0.25 from univariable analysis were initially included. A backwards‐stepwise approach then eliminated each variable manually until only those with a significant likelihood ratio (LRT) P‐value (≤0.05) remained, or where their removal substantially (>25%) altered coefficients of other variables indicating confounding. Each eliminated variable was individually added back into the multivariable model and the final model assessed for two‐way interactions to further establish best fit.

**Table 2:** Serology results, adjusted for clustering within site, for respiratory pathogens in working horses in Ethiopia (n = 350). Virus neutralisation testing for EAV of those positive on ELISA only (n = 17), Haemagglutination Inhibition testing only for EIV of potentially disputable negative ELISA results only (n = 20)

Pathogen	Test (cut off)	Seropositive (n)	Prevalence (%)	Lower 95% CI	Upper 95% CI
*S. equi*	ELISA (≥0.5)	33	8.0	4.7	13.1
EAV	ELISA (≥0.5)	17	4.2	2.1	8.3
	Virus neutralisation (≥75%)	1	0.3	<0.01	2.1
EIV	ELISA (≥55%)	1	0.3	<0.01	2.1
	Haemagglutination inhibition (≥1:32)	0	0.0		
EHV‐1	Complement fixation (≥1:80)	0	0.0		
EHV‐4	Complement fixation (≥1:80)	0	0.0		
ERAV	Complement fixation (≥1:80)	0	0.0		
ERBV	Complement fixation (≥1:80)	0	0.0		

EAV, equine arteritis virus; EIV, equine influenza virus (H7N7 Prague/56, H3N8 Miami/63 and H3N8 Newmarket/2/93); EHV, equine herpesvirus, ERA/BV, equine rhinitis virus; CI, confidence intervals.

The intra‐class correlation coefficient (ICC) for within‐site clustering was estimated using the latent‐variable method 22, which assumes variation at the individual level is approximately equal to (π^2^)/3.

## Results

**Figure 1:**
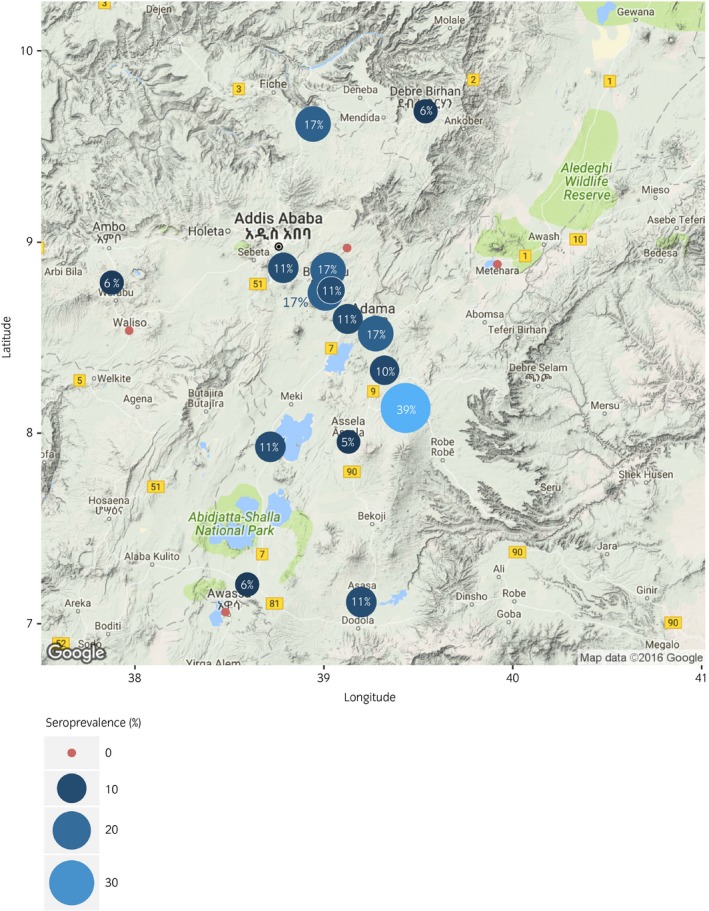
Map showing sampling sites and seroprevalence for *S. equi*. Red points indicate a site where no seropositive horses were detected. Blue points are proportional in size to the prevalence of *S. equi* positive animals detected at each location.

A total of 19 study locations (Fig 1) and 395 participants were selected, with 350 horses undergoing both clinical examination and blood sampling. Behaviour prohibited examination and blood sampling in 45 horses. The vast majority of participants (97%) owned the animal examined, with duration of ownership ranging from 2 days to 15 years (median 1.0 years, interquartile range (IQR) 0.5–3.0 years). Of these, over half (55%) owned only the horse examined whereas 39% owned a second horse. The majority (84%) were driven as ‘gharry’ carthorses, most citing their cart as their main source of income (83%). Median distance travelled to sample site was 2 km (IQR 1–5 km), but nearly 10% travelled over 10 km. Uptake of free vaccination for African Horse Sickness was approximately 30%, and owners reported that 43% of animals had received an antiparasitic treatment in the last 12 months.

The vast majority (95%) of animals examined were male, two‐thirds (70%) of which were gelded. Estimates of age from dentition suggest a median of 12 years (IQR 9–15 years), but animals ranged from 9 months to over 20 years old.

**Figure 2:**
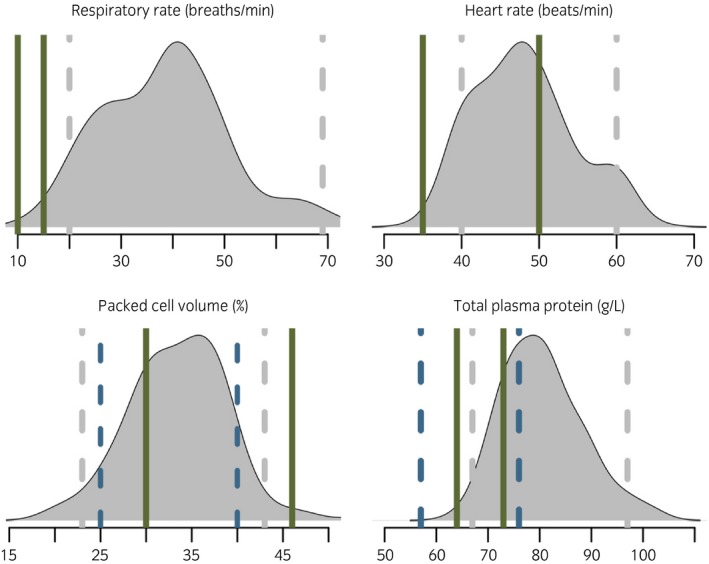
Smoothed distribution curves of clinical parameters for rested horses (n = 151), shown with comparison to published reference intervals for other populations. Upper and lower limits for normal for a UK horse population 23 (green). Comparison of haematological values to those from another working equid population in Pakistan (blue dashes) 24. 2.5 and 97.5% percentiles for this study population (grey dashes).

Most horses (82%) were in moderate (BCS 2/5) or good body condition (BCS 3/5), but nearly a fifth (18%) were in poor or very poor body condition (BCS 0–1/5). Owners reported a recent (preceding 30 days) history of coughing in 38% of cases (n = 134), nasal discharge in 7% (n = 25: 15 serous, 8 purulent, 2 bloody), while 3% (n = 11) reported recently suffering other breathing problems. No animals were noted to cough during clinical examination but 6% had an observed nasal discharge (n = 21: 10 serous, 9 mucopurulent, one bloody, one food material). Five animals showed enlarged submandibular lymph nodes but not in combination with nasal discharge or pyrexia. Increased lung sounds on auscultation were heard in 5% of horses. Median respiration rate (RR) was 40 breaths/min (IQR 32–48) and median heart rate (HR) was 48 beats/min (IQR 44–52). Median PCV was 34% (IQR 30–37) and TPP was 80 g/L (IQR 74–86). Due to the potential effects of recent exercise, values for animals resting for a minimum of 20 min prior to examination (n = 151) were also assessed but median values were not altered for this subset. Measurements for HR, RR, PCV and TPP were compared with ‘normal’ reference intervals (Fig 2) commonly used in the UK 23 and, where available, to working equid reference intervals 24. A large number of Ethiopian horses had parameters that were in excess of normal UK limits, particularly for RR (150/151 RR>15) and TPP (119/151, TPP>73).

### Serological results

The results of serological testing of all samples are presented in Table 2. Antibodies to *S. equi* were the most prevalent (8%). Previous exposure to equine arteritis virus was confirmed by VN in one animal (0.3%). Presence of low antibody titres in half of those animals tested by CFT provided evidence of prior exposure to EHV‐1/‐4 or ERA/BV in half of those tested but was not indicative of response to recent infection (Supplementary Item 3). Antibodies to EIV were not detected. *S. equi* prevalence by sampling location is shown in Figure 1 and Supplementary Item 4.

### Horse‐ and owner‐level associations with *S. equi* seropositve horses

The results of univariable logistic regression analysis of clinical examination findings and questionnaire responses with *S. equi* serostatus, adjusted for clustering within site, are presented in Supplementary Items [Link], [Link], [Link]. There were no significant associations between serostatus for *S. equi* and clinical examination findings (Supplementary Item 5).

To evaluate the shape of the relationship between continuous explanatory variables and the outcome, generalised additive models (GAM) were used. Age and number of days worked were linearly associated with the outcome. The final multilevel model showed that age of horse and number of days worked per week were significantly linearly associated with *S. equi* seropositive status (Table 3). The odds of seropositive status decreased by 0.9 (95% CI 0.81–0.96) for each year increase of a horse's age. Similarly, the odds of seropositive status decreased by 0.7 (95% CI 0.49–0.98) for each additional day worked per week, no significant interactions between variables remaining in the final model were detected.

**Table 3:** Multivariable, multilevel logistic regression model of factors associated with *S. equi* seropositive working horses in Ethiopia across 19 sites adjusted for within‐site clustering (n = 350)

Variable	Coefficient	Odds ratio	Lower 95% CI	Upper 95% CI	LRT P‐value
Dental age (per year increase in age)	−0.12	0.9	0.81	0.96	<0.01
Days worked per week (per day increase in time worked)	−0.36	0.7	0.49	0.98	0.04

LRT, likelihood ratio test.

From the final model, the ICC for *S. equi* within sampling location was estimated to be zero.

## Discussion

This is the most comprehensive study into the frequency of signs and pathogens associated with infectious respiratory disease in the working horses of Ethiopia. Results demonstrated an 8% seroprevalence to *S. equi*, the causative agent of strangles, similar to the prevalence reported in one other African study in Lesotho (10%) 10 that used similar diagnostic techniques (dual antigen ELISA) 25 and in Saudi Arabia (12%) 26. A positive ELISA result suggests prior exposure to *S. equi* causing active infection, persistent colonisation with carrier status, or immunity following a full recovery 27, 28.

There was no association between *S. equi* serostatus and clinical examination findings or a history of respiratory signs reported by owners. One potential explanation, as in other endemic areas 28, is that a milder, atypical form of the disease exists, with little or no nasal discharge and no discharging lymph nodes 29, 30. This presentation following infection can be linked with challenge dose (A. Waller, personal communication), previous exposure to *S. equi* or *S. zooepidemicus*, or host‐factors such as age or immunocompetence 28. The lack of association of serostatus with clinically apparent disease may also be a consequence of the cross‐sectional sero‐epidemiological study design, as detectable antibody levels arise only 2 weeks after infection and can persist for weeks or several months following resolution of signs 25. A second serum sample would normally be recommended to assess the dynamic nature of the titre but was not possible in this study design.

From analysis it was apparent that those animals working more frequently (greater number of days per week) might be at lower odds of being seropositive for *S. equi*. Rather than hypothesising a causal relationship leading to disease, this may be due to the effect of convalescent animals working less frequently, and as such it is difficult to establish the impact of work on serological status. There may also be some bias introduced by sampling at the horses’ place of work, leading to an over‐representation of those animals able to work more frequently and thus missing those subjects absent from work due to sickness (healthy‐worker bias). This may have led to an underestimation of disease prevalence in this study.

In this study, younger horses may be at greater odds of being seropositive to *S. equi* than older animals. Younger animals were perhaps more frequently exposed to infection, or older horses may have lower or shorter duration of antibody levels. The latter would be contrary to the expected response in immune animals following re‐exposure, where a greater, more rapid anamnestic B cell‐mediated immune response is normally mounted and is expected to last longer. Infection with *S. equi* also stimulates a mucosal response in the nasopharynx, but it is unclear if the organism could be cleared without a systemically detectable rise in antibodies 31. Thus, it could be proposed that significant differences in serostatus of horses of different ages do not represent a difference in exposure to the pathogen, but in host‐pathogen interactions. It is also possible that age could be a proxy for another, unmeasured exposure risk. Neither identified risk‐factor presents an easily modifiable solution that could be recommended from this work.

Clustering of *S. equi* serostatus was very low, however the sampling locations had large equine populations and some participants travelled long distances to the sample site, thus a low ICC is plausible.

Viral pathogens are frequently implicated in respiratory disease 32 but seroprevalence in this population was low. Findings are in contrast to earlier studies reporting the prevalence of antibodies to EHV‐1 and ‐4 in Ethiopian horses of 14–21% and 88–96% respectively 12, 33. However, these previous studies both used ELISA serological tests, and show similar EHV‐1 and ‐4 detection rates to studies using the same methodologies elsewhere 34. Thus, this discrepancy is likely due to use of complement fixation techniques in this study, with results possibly indicative of more recent infection where others had detected longer lasting virus neutralising antibodies 35. This apparently low rate of detection in Ethiopia compared with surveillance studies for EHV elsewhere may also, in part, be due to differences in sampling between studies. Prevalence of herpesvirus in other studies is often from animals either presenting with signs of respiratory disease 8, 9, or present at certain events (e.g. sales), more often attended by young naïve horses 11, 36.

Detection of equine arteritis virus was rare and therefore is unlikely to be a major contributor to respiratory disease in this population. However, EAV is an important cause of reproductive losses causing abortion, and the single confirmed positive animal in this survey was a breeding mare. It is difficult to speculate as to the significance of this finding as VN antibodies to EAV can last for years 36. Mares were underrepresented in the sample, and it appears that mares are more commonly found in breeding areas located in the highlands that may have a different exposure rate to EAV.

The single borderline positive result on ELISA testing for influenza antibodies was neither repeatable, nor confirmed by HI test. This may potentially indicate a novel strain/clade of influenza not targeted in HI testing (H3N8 nor H7N7), or it is more likely a false positive test result due to the detection limit of the test. The lack of circulating antibodies to EIV makes this population naïve and potentially vulnerable to outbreaks of equine influenza. There are few disease control measures in place in Ethiopia to mitigate this risk, but in the author's experience, the low value of horses and difficulty of transport currently means there is limited long‐distance or international horse movement, which may keep any risk of introduction low.

Coughing and abnormalities on respiratory auscultation were relatively rare on clinical examination in this study. This may be a limitation of the examination, due to it being performed rapidly under roadside conditions, to allow owners to return to work as soon as possible. In contrast, owner reporting of recent respiratory signs was more common and in agreement with findings from participatory studies 5, 6. There was some disagreement between veterinary clinical examination findings and owner questionnaire responses. For example, 21 (6%) animals were identified with a nasal discharge during examination, but only 6 of these were reported by participants in their questionnaire responses. Literature on the reliability of owner‐reporting of clinical signs suggests it is a fairly successful tool for chronic respiratory disease 37 but reporting of more general health problems by owners has been less useful 10, 38. To minimise bias arising from inter‐observer variation in subjective assessments, all examinations were carried out by the first author.

Analysis of some clinical parameters relied on established limits for normal animals; for example RR, HR, PCV and TPP 23, 24. However, a greater proportion of horses than expected fell outside these limits, with high RR and increased TPP levels in over three‐quarters of horses in this study. Interpretation of these values without further biochemical analysis is difficult. To avoid the impact of using these potentially inappropriate limits for normal cut‐offs, parameters were assessed as both continuous and categorical variables. The apparent deviation of this population from previously published ranges for other populations may suggest the need for further investigation.

Limitations of using a cross‐sectional study design with serological indication of pathogen‐exposure are that prevalence relates to both the frequency of infection but also the duration of the antibody response. The use of a single measure of antibody may over‐represent diseases with a longer duration of raised antibody, chronic disease or persistently infected carrier states. It also fails to account for infection in immunocompromised animals with a reduced antibody response, nor does it capture any seasonal pattern to disease, outbreaks of infection or reactivation of latent infections. An area not fully addressed in this current study was the identification of, and role played by, *S. equi* carrier animals as a source of new infections. This was partly due to the limited diagnostic capabilities in‐country, where the gold standard testing of guttural pouch lavage was not readily available. While this study was able to give some indication of co‐infections of respiratory pathogens, it was not possible to look at a range of infections potentially affecting overall health. This would be an area of interest for future work.

The results of this study highlight the presence of infectious causes of respiratory disease in the working horses in central Ethiopia. It appears that *S. equi* may be endemic in this working equid population, however further studies to assess the role of other infections and noninfectious respiratory disease would help to build a more complete picture of disease in this population.

## Authors’ declaration of interests

No competing interests have been declared.

## Ethical animal research

Ethical approval for this study was granted by the Ethics Committee at the University of Liverpool (VREC89a). Horse owners gave consent for their animal's inclusion in the study.

## Sources of funding

G. Laing’s studentship was funded by the Institute of Infection and Global Health, University of Liverpool. The project was funded by a grant from SPANA awarded to G. Pinchbeck and R. Christley.

## Authorship

G. Pinchbeck, R. Christley and A. Stringer conceived of the study, G. Laing, R. Christley and G. Pinchbeck designed the study. G. Laing, T. Ashine and N. Aklilu conducted fieldwork, G. Laing conducted laboratory analysis and drafted the manuscript. All authors contributed to writing the manuscript.

## Supporting information


**Supplementary Item 1**: Participant questionnaire (English Language version).

**Supplementary Item 2:** Nasal discharge chart used to aid participants’ description of respiratory signs seen in their working horses: 1‐none, 2‐serous, 3‐mild mucopurulent, 4‐unilateral mucopurulent, 5‐severe mucopurulent, 6‐epistaxis.

**Supplementary Item 3:** Antibody titre results from complement fixation for EHV‐1, EHV‐4, ERAV and ERBV. Anti‐complementary activity was included and low final titres indicate historical exposure but not recent infection.

**Supplementary Item 4:** *S. equi* serology results (positive ≥0.5 and borderline 0.3–0.4) in working horses in Ethiopia (n = 350) by sampling location (in order of elevation from high to low). Possible co‐infection is indicated by number of horses seropositive to *S. equi* and with low antibody titres to viral pathogens (EVA n = 0, EHV‐1 n = 14, EHV‐4 n = 16, ERAV n = 6, ERBV n = 10, EIV n = 0).

**Supplementary Item 5**: Univariable multilevel logistic regression analysis of clinical examination and haematological variables associated with serostatus for *S. equi* in working equids (n = 350). Odds ratios and confidence intervals are adjusted for within‐site clustering.

**Supplementary Item 6**: Univariable, multilevel logistic regression model of continuous risk factors and clinical parameters associated with *S. equi* seropositive working equids across 19 sites in Ethiopia, adjusted for within‐site clustering (n = 350).

**Supplementary Item 7:** Univariable, multilevel logistic regression model of risk factors associated with *S. equi* seropositive working equids across 19 sites in Ethiopia, adjusted for within‐site clustering (n = 350).

## Manufacturer's address

1 IDVet‐Innovative Diagnostics, Montpellier, France.
