# Supplementary material for: Respiratory disease and sero‐epidemiology of respiratory pathogens in the working horses of Ethiopia
Source: Equine Vet J. 2018 May 17;50(6):793–9. doi: 10.1111/evj.12834 (PMC6175379; doi:10.1111/evj.12834)
Supplement: Supplementary file 1 — Supplementary Item 1: Participant questionnaire (English Language version). [file EVJ-50-793-s001.pdf]

Supplementary Item 1:

Equine Veterinary Journal

SPANA
For the working animals of the world

ID No.	Date:	
	Location:	
	CHA Plate:	

Owner Questionnaire

1.	How long has your horse been resting ?	< 10 mins	10 - 30 mins	>30 mins			
2.	How far have you travelled from your home today?						
3.	*i)* Do you (or your family) own this horse?	Yes	No -hired	Other:			
	ii) How long have you worked with/owned **any** horses?	<1yr	1yr+				
4.	*i)* Does this horse pull a cart?	Yes	No (Saddle)	No (Other):			
	ii) Is your cart business your main source of income?	Yes	No				
5.	*i)* How long have you owned **this** horse?	*i)*	*ii)*				
	ii) How many horses do you own?						
6.	How many full or half days does **this** horse work in a week?	Full:	Half:				
7.	How old is this horse?	years	Unknown	Young	Adult	Old	
8.	Where do your horses come from? *(town/region)*						
9.	*i)* What do you feed your horse? (Circle)	Wheat Bran	Grains	Straw / hay	Grass	Other Feed:	
	ii) Is the feed soaked?	Yes & fed immed.	Yes & fed later	Sometimes	Never		
	iii) Do they share a feeding area with other horses?	Yes	No	Sometimes			
10.	How is your horse kept at night?	Turned Loose	Under shelter/ in compound alone	Shared shelter / compound			
11.	Has this horse had any nasal discharge in the last 30days? *If yes, describe & show chart (answer 1- 6)*		Chart no.	No discharge	Unknown		
12.	Has this horse been coughing in the last 30days? *If yes, when?*	No cough	All the time	At work	At rest/night	At feeding	Occasional	Unknown
13.	Have there been any other breathing problems in the last 30days?	None	Unknown				
14.	i) Has the horse had vaccination in the last 12months?	No Vaccination					
		Into mouth (deworming)	Injection into neck	*If known:*	AHS	Tetanus	
	ii) *Location:*	**SPANA**	Gov/Private Clinic	Self bought & admin	Traditional product		
