# Supplementary material for: Respiratory disease and sero‐epidemiology of respiratory pathogens in the working horses of Ethiopia
Source: Equine Vet J. 2018 May 17;50(6):793–9. doi: 10.1111/evj.12834 (PMC6175379; doi:10.1111/evj.12834)

Equine Veterinary Journal

Supplementary Item 2: Nasal discharge chart used to aid participant's description of respiratory signs seen in their working horses: 1-none, 2-serous, 3-mild mucopurulent, 4-unilateral mucopurulent, 5-severe mucopurulent, 6-epistaxis.

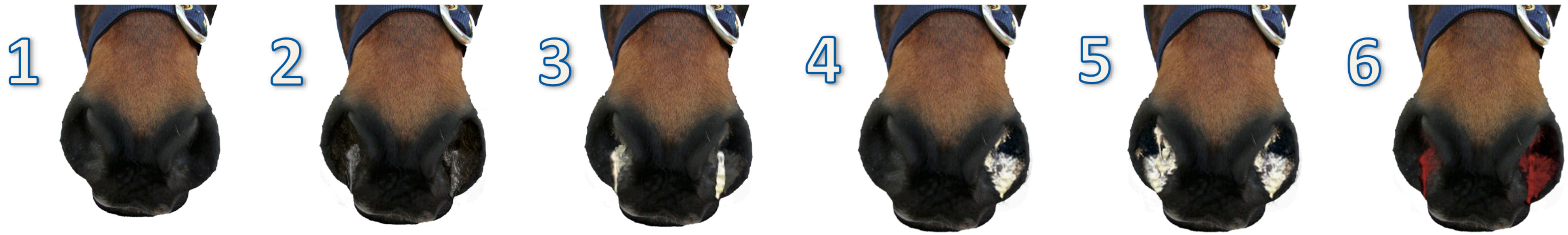

Supplement: Supplementary file 2 — Supplementary Item 2: Nasal discharge chart used to aid participants’ description of respiratory signs seen in their working horses: 1‐none, 2‐serous, 3‐mild mucopurulent, 4‐unilateral mucopurulent, 5‐severe mucopurulent, 6‐epistaxis. [file EVJ-50-793-s002.pdf]
