# Supplementary material for: Respiratory disease and sero‐epidemiology of respiratory pathogens in the working horses of Ethiopia
Source: Equine Vet J. 2018 May 17;50(6):793–9. doi: 10.1111/evj.12834 (PMC6175379; doi:10.1111/evj.12834)
Supplement: Supplementary file 3 — Supplementary Item 3: Antibody titre results from complement fixation for EHV‐1, EHV‐4, ERAV and ERBV. Anti‐complementary activity was included and low final titres indicate historical exposure but not recent infection. [file EVJ-50-793-s003.pdf]

Equine Veterinary Journal

Supplementary Item 3: Antibody titre results from complement fixation for EHV-1, EHV-4, ERAV and ERBV. Anti-complementary activity was included and low final titres indicate historical exposure but not recent infection.

	Antibody titre (n)				
	**1:5**	**1:10**	**1:20**	**1:40**	**Total**
Equine herpesvirus -1 (EHV-1)	75	12	1	0	88
Equine herpesvirus -4 (EHV-4)	75	27	6	1	109
Equine rhinitis A virus (ERAV)	34	9	5	0	48
Equine rhinitis B virus (ERBV)	68	11	4	3	86
