# Supplementary material for: Respiratory disease and sero‐epidemiology of respiratory pathogens in the working horses of Ethiopia
Source: Equine Vet J. 2018 May 17;50(6):793–9. doi: 10.1111/evj.12834 (PMC6175379; doi:10.1111/evj.12834)
Supplement: Supplementary file 4 — Supplementary Item 4: S. equi serology results (positive ≥0.5 and borderline 0.3–0.4) in working horses in Ethiopia (n = 350) by sampling location (in order of elevation from high to low). Possible co‐infection is indicated by number of horses seropositive to S. equi and with low antibody titres to viral pathogens (EVA n = 0, EHV‐1 n = 14, EHV‐4 n = 16, ERAV n = 6, ERBV n = 10, EIV n = 0). [file EVJ-50-793-s004.pdf]

Equine Veterinary Journal

Supplementary Item 4: *S equi* serology results (positive ≥0.5 and borderline 0.3-0.4) in working horses in Ethiopia (n=350) by sampling location (in order of elevation from high to low). Possible co-infection is indicated by number of horses seropositive to *S equi* that also had low antibody titres to viral pathogens (EVA n=0, EHV-1 n=14, EHV-4 n=16, ERAV n=6, ERBV n=10, EIV n=0)

Town code	Sampling Location (town)	Agroclimatic Zone	Elevation (m)	n	*S equi* (≥0.5)	Borderline (0.3-0.4)	Prevalence (95% CI)	Animals with titres to other pathogens (n, pathogen)
WEN	Wenchi	Highland	3115	18	1	1	5.6% (1.0-27.6)	1 (EHV-1 + 4)
DB	Debre Brehan	(>2300m)	2834	18	1	5	5.6% (1.0-27.6)	1 (EHV-1)
SAL	Salali		2643	18	3	4	16.7% (5.8-39.2)	2 (EHV-4)
CHE	Chefe		2424	18	0	0		
ASEL	Asella		2399	19	1	5	5.3% (0.9-24.6)	
ASA	Asasa		2360	18	2	5	11.1% (3.1-32.8)	1 (EHV-4)
AK	Akaki	Midland	2036	18	2	5	11.1% (3.1-32.8)	
GOD	Godino	(1500-2300m)	2010	18	3	2	16.7% (5.8-39.2)	1 (EHV-1+4, ERAV), 1 (EHV-1+4, ERA/BV)
WOL	Woliso		1929	20	0	2		
SHA	Shashemene		1920	18	1	3	5.6% (1.0-27.6)	
ARB	Arb Gebeya		1887	18	7	0	38.9% (20.3-61.4)	4 (EHV-1+4, ERA/BV), 1 (EHV-1+4, ERBV), 1 (EHV1+4)
DZ	Debre Zeit		1803	18	2	2	11.1% (3.1-32.8)	1 (EHV-1+4, ERA/BV), 1 (EHV-1+4)
DZO	D.Z out of town		1803	18	3	1	16.7% (5.8-39.2)	1 (EHV-1+4, ERA/BV), 1 (EHV-4)
MOJ	Modjo		1785	19	2	6	10.5% (2.9-31.4)	1 (EHV-1)
HAW	Hawassa		1702	18	0	0		
DE	Dera		1671	20	2	0	10.0% (2.8-30.1)	
ZWY	Ziway		1653	18	2	2	11.1% (3.1-32.8)	1 (EHV-4)
AD	Adama		1595	18	3	3	16.7% (5.8-39.2)	1 (EHV-4)
MET	Meteharra	Lowland (<1500m)	955	20	0	0		

CI – Confidence Interval
