# Supplementary material for: Respiratory disease and sero‐epidemiology of respiratory pathogens in the working horses of Ethiopia
Source: Equine Vet J. 2018 May 17;50(6):793–9. doi: 10.1111/evj.12834 (PMC6175379; doi:10.1111/evj.12834)
Supplement: Supplementary file 5 — Supplementary Item 5: Univariable multilevel logistic regression analysis of clinical examination and haematological variables associated with serostatus for S. equi in working equids (n = 350). Odds ratios and confidence intervals are adjusted for within‐site clustering. [file EVJ-50-793-s005.pdf]

Equine Veterinary Journal

Supplementary Item 5: Univariable multilevel logistic regression analysis of clinical examination and hematological variables associated with serostatus for *S equi* in working equids (n=350). Odds ratios and confidence intervals are adjusted for within-site clustering. Some variables were considered as both categorical and continuous responses (Supplementary Item 6) to avoid difficulties in allocating reference intervals for 'normal'. Variables with empty cells had a single positive result added to allow calculation of odds ratio.

		Serology results for S equi ≥0.5					
**Variable**		**+ (%)**	**- (%)**	**Odds ratio**	**Lower 95% CI**	**Upper 95% CI**	***Wald p*-value**
**Signalment**							
Sex	gelding	18 (55)	217(68)	ref			
	stallion	11 (33)	87 (27)	1.4	0.6	3.3	0.4
	mare	4 (12)	13 (4)	2.4	0.5	11.9	0.3
**Clinical Examination**							
Nasal discharge	absent	32 (97)	293 (94)	ref			
	present	1 (3)	20 (6)	0.4	<0.01	2.9	0.3
Cranial lymph nodes	normal	33 (100)	310 (98)	ref			
	enlarged	0	5 (2)	1.9	0.2	18.6	0.6
Respiratory	normal	33 (100)	299 (94)	ref			
auscultation	abnormal	0	18 (6)	0.7	0.1	5.4	0.7
Pyrexia	<39.0°C	32 (97)	313 (99)	ref			
	≥39.0°C	1 (3)	4 (1)	2.7	0.2	29.5	0.4
Body condition score	underweight (<2)	4 (12)	60 (19)	0.6	0.2	1.9	0.4
(0-5)	moderate/good	29 (88)	257 (81)	ref			
	overweight (>3)	0	0	-			
**Haematology**							
Packed cell volume	<30%	2 (6)	46 (15)	0.4	0.1	2.0	0.3
	30-46%	31 (94)	265 (84)	ref			
	>46%	0	6 (2)	2.0	0.2	19.6	0.6
Total plasma protein	<6.5 g/L	0	0	-			
	6.5-7.3 g/L	2 (6)	46 (15)	ref			
	>7.3 g/L	31 (94)	271 (85)	3.7	0.8	17.3	0.1

CI - Confidence interval, ref- Reference category
