# Supplementary material for: Respiratory disease and sero‐epidemiology of respiratory pathogens in the working horses of Ethiopia
Source: Equine Vet J. 2018 May 17;50(6):793–9. doi: 10.1111/evj.12834 (PMC6175379; doi:10.1111/evj.12834)
Supplement: Supplementary file 6 — Supplementary Item 6: Univariable, multilevel logistic regression model of continuous risk factors and clinical parameters associated with S. equi seropositive working equids across 19 sites in Ethiopia, adjusted for within‐site clustering (n = 350). [file EVJ-50-793-s006.pdf]

Equine Veterinary Journal

Supplementary Item 6: Univariable, multilevel logistic regression model of continuous risk factors and clinical parameters associated with *S equi* seropositive working equids across 19 sites in Ethiopia, adjusted for within-site clustering (n=350).

	**Serology results for *S equi* ≥0.5**					
	**+**	**-**				
**Variable**	**Median (IQR)**	**Median (IQR)**	**Odds ratio**	**Lower 95% CI**	**Upper 95% CI**	***Wald p*-value**
Dental Age	10 (7-13)	12 (9-15)	0.9	0.8	0.9	<0.01*
Respiration rate (br/min)	40 (32-44)	40 (32-48)	1.0	1.0	1.0	0.3
Heart rate (bpm)	48 (40-56)	48 (44-52)	1.0	0.9	1.0	0.6
PCV (%)	34 (30-38)	34 (30-37)	1.0	0.9	1.1	0.8
TPP (g/L)	8.0 (7.6-8.2)	8.0 (7.6-8.6)	0.9	0.5	1.7	0.8
Days worked/week	2.0 (1.0-3.5)	3.0 (2.5-3.5)	0.6	0.4	0.9	0.0*
Distance travelled to site (km)	3 (1-8)	2 (1-5)	1.1	1.0	1.2	0.2*

CI Confidence Interval, IQR – Interquartile Range, *variables taken through to multivariable analysis
Nb. participants described using two horses over the course of a day's work (hence each horse completing half a day), where a half-day was described as 6 hours. Hence median 'Days worked/week' was often the sum of multiple half days.
