# Supplementary material for: Respiratory disease and sero‐epidemiology of respiratory pathogens in the working horses of Ethiopia
Source: Equine Vet J. 2018 May 17;50(6):793–9. doi: 10.1111/evj.12834 (PMC6175379; doi:10.1111/evj.12834)
Supplement: Supplementary file 7 — Supplementary Item 7: Univariable, multilevel logistic regression model of risk factors associated with S. equi seropositive working equids across 19 sites in Ethiopia, adjusted for within‐site clustering (n = 350). [file EVJ-50-793-s007.pdf]

Equine Veterinary Journal

Supplementary Item 7: Univariable, multilevel logistic regression model of risk factors associated with *S equi* seropositive working equids across 19 sites in Ethiopia, adjusted for within-site clustering (n=350).

Variable		***Serology results for S equi ≥0.5***					
		**+ (%)**	**- (%)**	**Odds ratio**	**Lower 95% CI**	**Upper 95% CI**	***Wald p*-value**
**Ownership & Work**							
Horse use	cart (gharry)	23 (70)	273 (86)	ref			
	saddle	10 (30)	43 (14)	2.4	0.9	6.5	0.1*
Ownership	hired	1 (3)	8 (3)	ref			
	owned	32 (97)	309 (97)	0.7	0.1	6.6	0.8
Duration owned	≤1month	2 (6)	25 (8)	1.0	0.2	5.1	>0.9
	≤1year	9 (28)	68(22)	1.6	0.7	4.0	0.3
	>1year	21 (66)	216 (70)	ref			
Owner eq. experience	≤1 year	4 (12)	45 (14)	ref			
	>1 year	29 (88)	272 (86)	1.2	0.4	3.8	0.7
Cart horse income:	not main source	15 (45)	88 (28)	ref			
	main income source	18 (55)	228 (72)	0.5	0.2	1.3	0.2*
No. horses owned	1	19 (59)	164 (54)	ref			
	2 or more	13 (41)	141 (46)	0.7	0.3	1.6	0.4
**Harnessing**							
Chest strap/	correct fitting	16 (48)	185 (59)	ref			
throat-lash/girth	tight fitting	17 (52)	129 (41)	1.7	0.8	3.6	0.2*
**Husbandry**							
Feeding:	no wheat bran	11 (33)	54 (17)	ref			
	wheat bran	22 (67)	262 (83)	0.5	0.2	1.3	0.2*
	no grains	18 (55)	181 (57)	ref			
	grains	15 (45)	136 (43)	0.9	0.4	2.1	0.8
	no hay/straw	15 (45)	124 (39)	ref			
	hay/straw	18 (55)	193 (61)	0.8	0.3	2.1	0.7
	no grass	18 (55)	183 (58)	ref			
	grass (grazed or cut)	15 (45)	134 (42)	1.1	0.4	2.6	0.9
Feed soaked	never	12 (36)	112(37)	ref			
before fed:	always	20 (61)	183 (61)	1.7	0.6	5.3	0.3
	variable	1 (3)	15 (2)	1.0	0.1	9.7	>0.9
Housing overnight:	individual	19 (58)	177 (56)	ref			
	shared	14 (42)	137 (43)	0.9	0.4	2.0	0.8
	turned loose	0	3 (1)	3.4	0.3	39.3	0.3
**Vaccination & Deworming**							
AHS vaccination	none	24 (73)	234 (74)	ref			
(last 12m)	vaccinated	9 (27)	81 (26)	1.0	0.4	2.6	>0.9
Deworming	none	16 (48)	182 (58)	ref			
(last 12m)	dewormed	17 (52)	134 (42)	1.3	0.5	2.9	0.6
**Clinical History (last 30d)**							
Nasal discharge	none	31 (94)	292 (93)	ref			
	present	2 (6)	23 (7)	0.7	0.1	3.3	0.6
Cough	none	19 (58)	197 (62)	ref			
	yes	14 (42)	120 (38)	1.1	0.5	2.3	0.9
Other breathing	none	32 (97)	299 (97)	ref			
problem	yes	1 (3)	10 (3)	1.1	0.1	10.3	0.9

CI- Confidence interval
